# Association between resting heart rate and low natural killer cell activity: a cross-sectional study

**DOI:** 10.3389/fimmu.2024.1465953

**Published:** 2024-09-27

**Authors:** Hyoju Oh, A-Ra Cho, Joo-Hwan Jeon, Eunkyung Suh, Junhyung Moon, Baek Hwan Cho, Yun-Kyong Lee

**Affiliations:** ^1^ Chaum Life Center, CHA University, Seoul, Republic of Korea; ^2^ Department of Family Medicine, Yonsei University College of Medicine, Seoul, Republic of Korea; ^3^ Department of Biomedical Informatics, CHA University School of Medicine, CHA University, Seongnam, Republic of Korea

**Keywords:** RESTING HEART RATE, cortisol, natural killer cell, natural killer cell activity, immunity

## Abstract

Resting heart rate (RHR), a simple physiological indicator, has been demonstrated to be associated with inflammation and even metabolic disorders. This study aimed to investigate whether RHR is associated with natural killer cell activity (NKA) in a large population of healthy adults using a novel assay to measure NKA. This cross-sectional study included 7,500 subjects in the final analysis. NKA was estimated by measuring the amount of interferon-gamma (IFN-γ) released by activated natural killer cells; low NKA was defined as IFN-γ level <500 pg/mL. Subjects were categorized into four groups according to RHR as follows: C1 (≤ 60 bpm), C2 (60–70 bpm), C3 (70–80 bpm), and C4 (≥ 80 bpm). Individuals with higher RHR exhibited poorer metabolic and inflammatory profiles, with the prevalence of low NKA being highest in the highest RHR category. Compared with C1 as reference, the fully adjusted odd ratios (ORs) [95% confidence intervals (CIs)] for low NKA were significantly higher in C3 (OR: 1.37, 95% CI: 1.08–1.75) and C4 (OR: 1.55, 95% CI: 1.20–2.00). In addition, RHR was shown to exert indirect effects on NKA upon consideration of the mediation effect of serum cortisol in path analysis. Our findings confirm a significant link between elevated RHR and low NKA, and suggest the usefulness of RHR, a simple indicator reflecting increased sympathetic nervous system activity and stress, in predicting reduced immune function.

## 1 Introduction

Resting heart rate (RHR) is a key physiological indicator that reflects the intrinsic pacemaker activity of the sinoatrial node simply measured by reliable methods like manual palpation or electrocardiography (1, 2). This simple yet critical vital sign is influenced by a myriad of factors, including autonomic nervous system activity (3), various hormonal regulations (4–6), age (7), fitness level (8), and overall cardiovascular health (9). Elevated RHR has been associated with a higher level of inflammation (10) and an increased risk of cardiovascular diseases, including hypertension (11), heart failure (12), atrial fibrillation (13), diabetes mellitus (14), metabolic syndrome (15), and even cognitive function (16), making it a valuable prognostic marker in clinical settings. Conversely, a lower RHR is often indicative of enhanced cardiovascular fitness and improved autonomic balance (17).

Natural Killer (NK) cells, key players in innate immunity, swiftly identify and eliminate virus-infected or abnormal cells without prior sensitization (18). Derived from bone marrow progenitors expressing CD56 and CD16, NK cells modulate immune responses through recognition of distressed cells (19). Their rapid response is crucial for early defense against infections and tumors, achieved via the release of cytotoxic granules and production of cytokines like interferon-gamma (IFN-γ) (20). NK cells influence adaptive immunity by interacting with dendritic cells and T cells, regulating antigen presentation and T cell balance which this intricate crosstalk enhances overall immune responsiveness and contributes to immune homeostasis (21). Reduced NK cell activity (NKA) has been associated with an increased susceptibility to infections in immunologically intact individuals and increased risk of various types of cancer development (22–24).

Previous studies have established that autonomic nervous system has a critical role in mediating interactions between the nervous and immune systems (25). Moreover, heart rate variability (HRV), reflecting the balance between the activities of the sympathetic and parasympathetic nervous systems, has been demonstrated to be associated with inflammation and infection (26). While majority of studies focus on the potential of HRV as a predictive marker of immunity (26, 27), there is no extensive body of research specifically linking between RHR, the simplest physiological indicator, and immune function. Highlighting the crucial role of NK cells in immune function, the aim of this study is to conduct an investigation whether resting heart rate is associated with NKA in a large population of healthy adults using a novel assay to measure NKA.

## 2 Materials and methods

### 2.1 Study population

**Figure 1:**
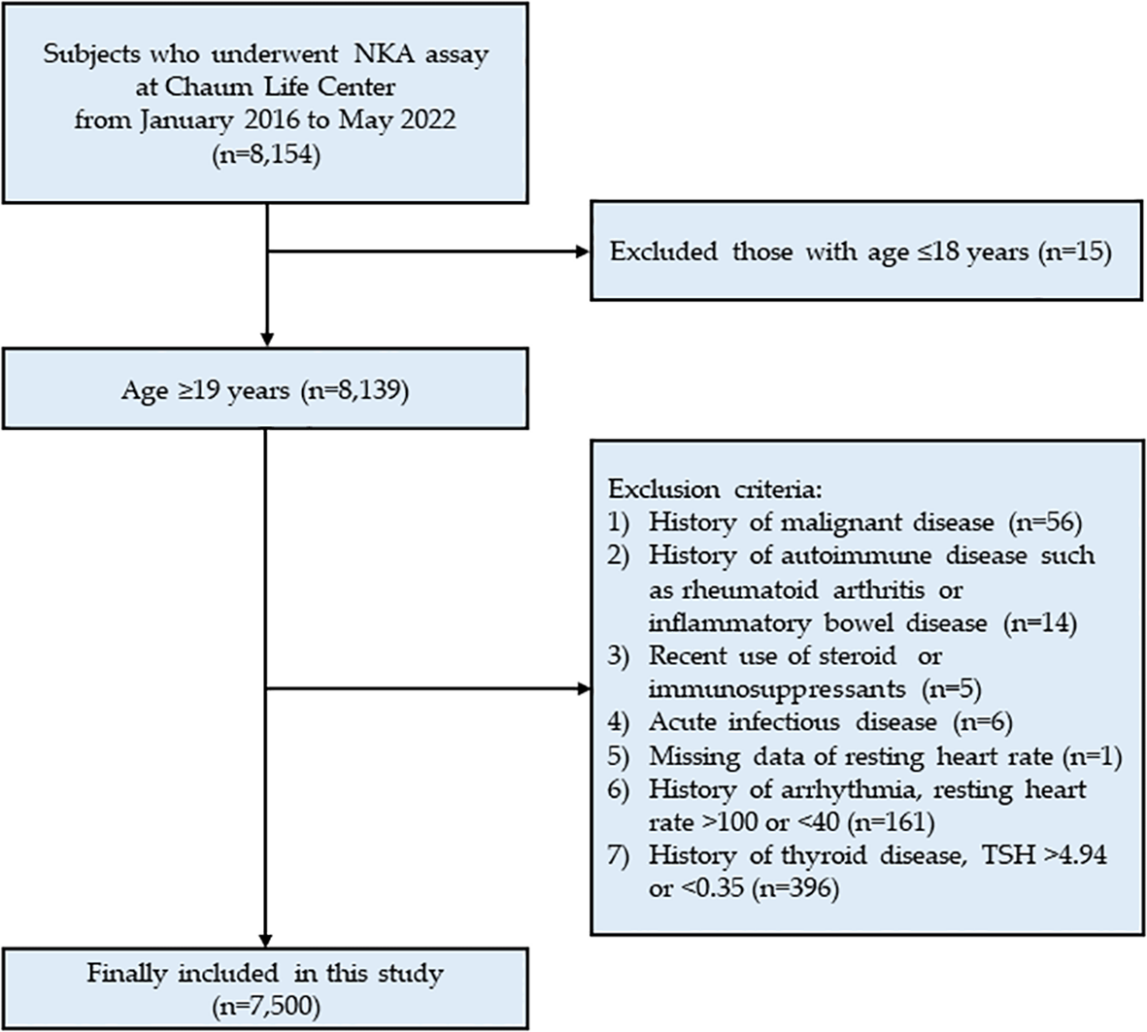
Flow chart of study population.

This cross-sectional study analyzed data from Chaum Life Center, screening subjects who underwent the NKA assay between January 2016 and May 2022 (n = 8,154). The data were obtained from electronic medical records. Among the 8,154 eligible subjects, 15 subjects under 18 years of age were excluded. Further exclusions were made for 1) those with a history of malignant disease (n = 56); 2) those with a history of autoimmune disease such as rheumatoid arthritis or inflammatory bowel disease (n = 14); 3) recent use of steroids or immuno-suppressants (n = 5); 4) acute infectious disease (n = 6); 5) those with missing data on resting heart rate (n = 1); 6) those with a history of arrhythmia, resting heart rate > 100 or < 40 bpm (n = 161); and 7) those with a history of thyroid disease, thyroid stimulating hormone (TSH) > 4.94 or < 0.35 mIU/L (n = 396). Ultimately, a total of 7,500 subjects were included in the analysis (**Figure 1**). The study protocol was approved by the Institutional Review Board of CHA Bundang Medical Center (CHAMC 2020-10-006). The study was conducted in accordance with the principles of the Declaration of Helsinki.

### 2.2 Data collection

Blood pressure (mmHg) was measured after at least 5 minutes of rest in a seated position. Systolic blood pressure (SBP) and diastolic blood pressure (DBP) were determined by averaging the last two of three measurements, with a minimum interval of 1 minute between each reading. Resting heart rate (RHR) was recorded from the right radial artery over a 60-second period. Blood samples were collected from the antecubital vein, in the morning after at least 8 hours of fasting. White blood cell (WBC) counts were quantified with XN-10 Hematology Analyzer (Sysmex, IL, USA). C-reactive protein (CRP) and fasting glucose were measured with the Hitachi 7600 Analyzer (Hitachi Co., Tokyo, Japan). Fasting insulin was measured by an electrochemiluminescence immunoassay using an Elecsys 2010 instrument (Roche, Mannheim, Germany). Insulin resistance was estimated using the homeostasis model assessment of insulin resistance (HOMA-IR) method by applying the following formula: HOMA-IR = fasting insulin (μIU/mL) × fasting glucose (mg/dL)/405 (28).

### 2.3 IFN-γ measurement for NKA

The IFN-γ released by activated NK cells was measured using a recently developed blood test (NK Vue^®^ Kit, NKMAX, Sungnam, Korea). A 1 mL sample of whole blood was transferred directly into a specific blood collection tube containing a patented stimulatory cytokine for NKA analysis (Promoca^®^, NKMAX, Sungnam, Korea). The collection tube was gently and repeatedly mixed within 30 minutes of collection and then incubated for 20–24 hours in a 37.0°C chamber, following the manufacturer’s instructions. During incubation, the stimulatory cytokine induces IFN-γ secretion into the plasma. Although only NK cells secrete detectable amount of IFN-γ after Promoca^®^ stimulation (29), interactions among activated immune cells might induce T cells and NKT cells to secrete IFN-γ. However, previous feasibility experiments have confirmed that the IFN-γ secretion predominantly occurs through NK cells rather than other innate or adaptive immune cells (29–31). After incubation, the supernatant was obtained and centrifuged at 3000 × g for 3 minutes. The supernatant was then loaded onto enzyme-linked immunosorbent assay (ELISA) plates, and the IFN-γ level was measured in pg/mL using the ELISA. Low NKA was defined as an IFN-γ level of less than 500 pg/mL (24, 32).

### 2.4 Statistical analysis

All data were presented as means ± standard deviations for continuous variables and as numbers (percentages) for categorical variables. Differences in clinical characteristics between participants with normal and low NKA were analyzed using the independent t-test for continuous variables and the chi-square test for categorical variables. Participants were categorized into four groups according to RHR as follows: C1 (≤ 60 bpm), C2 (60–70 bpm), C3 (70–80 bpm), and C4 (≥ 80 bpm). Comparisons of clinical characteristics among RHR categories were assessed using analysis of variance (ANOVA) for continuous variables and the chi-squared test for categorical variables. *Post hoc* analysis with the Bonferroni correction was conducted to determine the specific pairs of categories exhibiting significant differences after identifying significant differences among the categories. Multivariable logistic regression analyses were performed to calculate the odds ratios (ORs) and 95% confidence intervals (CIs) for low NKA, adjusting for age and sex in model 2, and for age, sex, DBP, WBC, CRP, and HOMA-IR in model 3. Additionally, path analysis was conducted to investigate the mediation effect of cortisol on NKA using the R statistical package (version 4.1.1; R Foundation for Statistical Computing, Vienna, Austria). The level of statistical significance for all analyses was set at p-value < 0.05. Statistical analyses were performed using SPSS statistical software (version 25.0, SPSS Inc., Chicago, IL, USA).

## 3 Results

### 3.1 Clinical characteristics of study populations

**Table 1** presents the clinical characteristics of study population. Age, SBP, DBP, RHR, WBC counts, and CRP levels were significantly higher in subjects with low NKA than those with normal NKA. No significant differences in proportion of male sex, glucose, insulin, HOMA-IR, and proportion of participants with hypertension, diabetes, and dyslipidemia were observed between subjects with normal and low NKA.

**Table 1:** Clinical characteristics of the study population.

Variables	Total	Normal NKA	Low NKA	*p* ^*^
Participants, n	7,500	4,743	2,757	
Age, years	48.0 ± 11.6	47.7 ± 11.7	48.6 ± 11.6	0.001
Male sex, n (%)	3,476 (46.3)	2,172 (45.8)	1,304 (47.3)	0.208
SBP, mmHg	119.0 ± 13.7	118.4 ± 13.6	120.0 ± 13.9	< 0.001
DBP, mmHg	77.0 ± 11.0	76.6 ± 10.8	77.7 ± 11.1	< 0.001
RHR, bpm	73.3 ± 10.3	72.8 ± 10.2	74.2 ± 10.3	< 0.001
WBC, cells/μL	5.40 ± 1.50	5.19 ± 1.35	5.74 ± 1.67	< 0.001
CRP, mg/dL	0.17 ± 0.42	0.14 ± 0.28	0.20 ± 0.59	< 0.001
Glucose, mg/dL	89.8 ± 17.7	89.8 ± 17.6	89.8 ± 18.0	0.983
Insulin, μU/mL	6.3 ± 5.0	6.3 ± 5.2	6.4 ± 4.8	0.469
HOMA-IR	1.49 ± 1.54	1.48 ± 1.59	1.50 ± 1.43	0.590
IFN-γ, pg/mL	1148.4 ± 1002.3	1685.8 ± 889.5	223.9 ± 141.7	< 0.001
Hypertension, n (%)	1031 (13.7)	651 (13.7)	380 (13.8)	0.233
Diabetes 3mellitus, n (%)	385 (5.1)	233 (4.9)	152 (5.5)	0.135
Dyslipidemia, n (%)	995 (13.3)	633 (13.3)	362 (13.1)	0.215

Data are presented as mean ± standard deviation or number (%). ^*^ *p*-value was calculated to compare the clinical characteristics between subjects with normal NKA and those with low NKA.

CRP, C-reactive protein; DBP, diastolic blood pressure; HOMA-IR, homeostatic assessment model of insulin resistance; RHR, resting heart rate; SBP, systolic blood pressure; WBC, white blood cell.

### 3.2 Comparison of clinical characteristics among RHR categories

**Table 2** shows the comparison of clinical characteristics among RHR categories using *post-hoc* analyses. All variables, except SBP, showed statistically significant differences among the four categories (overall *p*-value < 0.05). Category 1 (RHR ≤ 60) had the lowest mean DBP, WBC counts, and HOMA-IR, and the highest mean age, IFN-γ levels, and proportion of male sex. Category 4 (RHR ≥ 80) had the highest mean DBP, WBC counts, CRP, insulin, and HOMA-IR, and the lowest mean age, IFN-γ levels, and proportion of male sex.

**Table 2:** Comparison of clinical characteristics among resting heart rate categories.

Variables	Category 1	Category 2	Category 3	Category 4	1 vs 2	1 vs 3	1 vs 4	2 vs 3	2 vs 4	3 vs 4	Overall
	(RHR ≤ 60)	(RHR 60–70)	(RHR 70–80)	(RHR ≥ 80)							
Participants, n	715	2,469	2,495	1,821							
Age, years	50.2 ± 12.1	49.5 ± 11.4	47.6 ± 11.5	45.7 ± 11.4	0.678	< 0.001	< 0.001	< 0.001	< 0.001	< 0.001	< 0.001
Male sex, n (%)	429 (60.0)	1,186 (48.0)	1,071 (42.9)	790 (43.4)	< 0.001	< 0.001	< 0.001	< 0.001	0.003	0.765	< 0.001
SBP, mmHg	118.2 ± 14.1	118.6 ± 14.0	119.3 ± 13.7	119.3 ± 13.3	1.000	0.402	0.420	0.439	0.512	1.000	0.088
DBP, mmHg	73.6 ± 11.3	75.8 ± 10.9	77.5 ± 10.9	79.2 ± 10.5	< 0.001	< 0.001	< 0.001	< 0.001	< 0.001	< 0.001	< 0.001
RHR, bpm	56.5 ± 3.4	66.0 ± 2.8	75.2 ± 2.8	87.3 ± 5.2	< 0.001	< 0.001	< 0.001	< 0.001	< 0.001	< 0.001	< 0.001
WBC, cells/μL	5.21 ± 1.33	5.24 ± 1.39	5.41 ± 1.48	5.66 ± 1.67	1.000	0.009	< 0.001	0.001	< 0.001	< 0.001	< 0.001
CRP, mg/dL	0.12 ± 0.20	0.15 ± 0.28	0.17 ± 0.47	0.22 ± 0.56	1.000	0.211	< 0.001	0.821	< 0.001	0.007	< 0.001
Glucose, mg/dL	89.2 ± 15.2	88.8 ± 14.7	90.7 ± 19.5	90.0 ± 19.8	1.000	0.254	1.000	0.001	0.144	1.000	0.001
Insulin, μU/mL	5.1 ± 3.5	5.9 ± 4.3	6.4 ± 4.7	7.2 ± 6.5	0.006	< 0.001	< 0.001	0.019	< 0.001	< 0.001	< 0.001
HOMA-IR	1.2 ± 0.9	1.4 ± 1.1	1.5 ± 1.5	1.7 ± 2.1	0.060	< 0.001	< 0.001	0.002	< 0.001	0.011	< 0.001
IFN-γ, pg/mL	1316.4 ± 1063.7	1170.0 ± 996.8	1154.1 ± 1002.8	1045.4 ± 973.4	0.003	0.001	< 0.001	1.000	< 0.001	0.003	< 0.001
Hypertension, n (%)	104 (14.5)	363 (14.7)	344 (13.8)	220 (12.1)	0.051	< 0.001	< 0.001	0.055	< 0.001	0.016	< 0.001
Diabetes mellitus, n (%)	27 (3.8)	94 (3.8)	162 (6.5)	102 (5.6)	0.052	< 0.001	< 0.001	< 0.001	< 0.001	0.034	< 0.001
Dyslipidemia, n (%)	87 (12.2)	333 (13.5)	343 (13.7)	232 (12.7)	0.041	< 0.001	< 0.001	0.103	< 0.001	0.042	< 0.001

Data are presented as mean ± standard deviation or number (%). Overall p-values were calculated using the analysis of variance (ANOVA) for continuous variables and chi-squared test for categorical variables.

CRP, C-reactive protein; DBP, diastolic blood pressure; HOMA-IR, homeostatic assessment model of insulin resistance; RHR, resting heart rate; SBP, systolic blood pressure; WBC, white blood cell.

**Figure 2:**
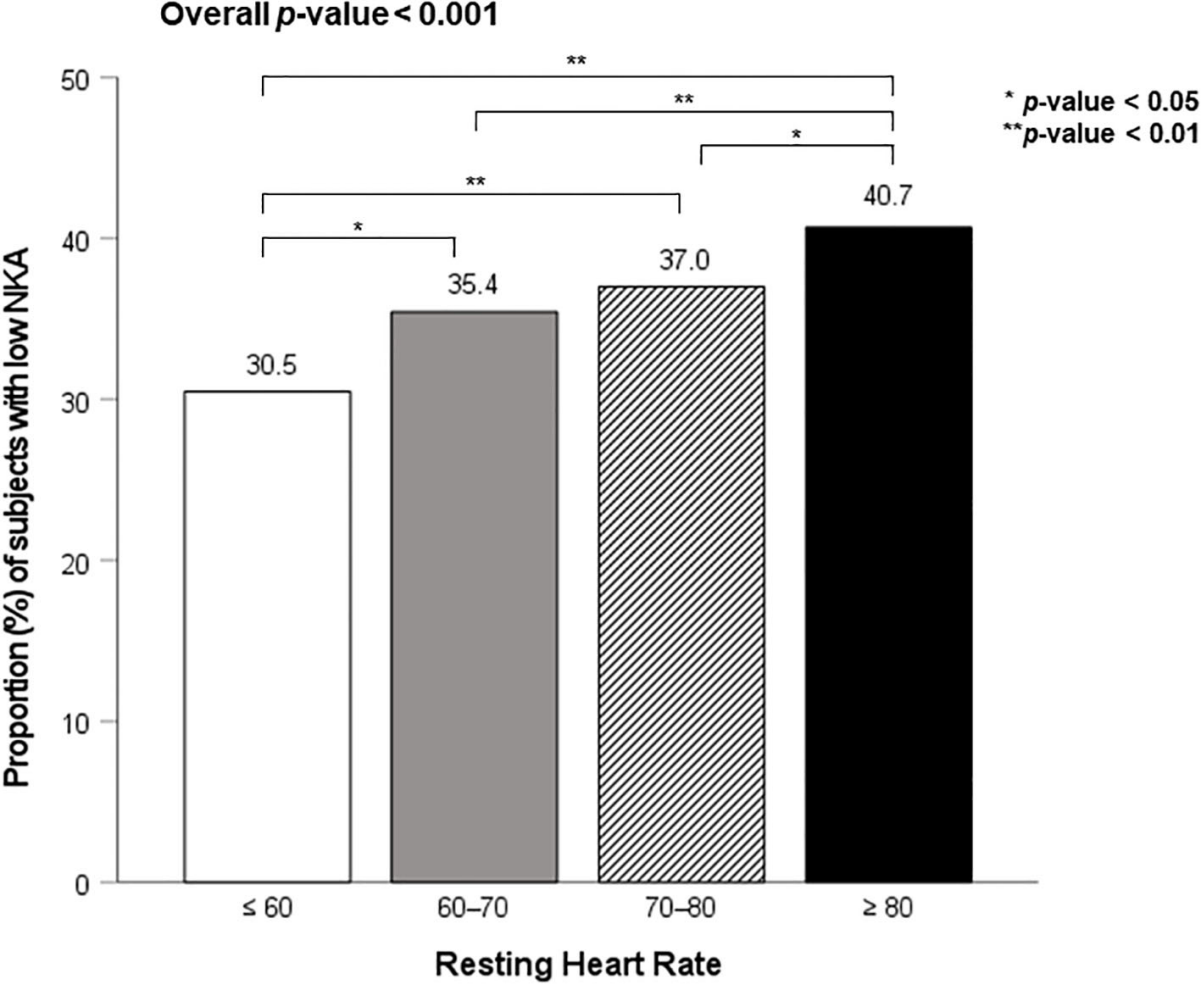
Proportion of subjects with low NKA according to RHR categories.

**Figure 2** shows the proportion of subjects with low NKA according to four RHR categories. The prevalence of low NKA was 30.5%, 35.4%, 37.0%, and 40.7% in C1, C2, C3, and C4, respectively. The prevalence of low NKA was significantly higher in C4, compared to C1, C2, and C3.

### 3.3 Association between RHR categories and low NKA

**Table 3** presents the results of multivariable logistic regression analysis of the relationship between RHR categories and low NKA. The unadjusted ORs (95% CIs) for low NKA of C2, C3, and C4 compared with C1 reference were 1.25 (1.05–1.50), 1.34 (1.12–1.60), and 1.56 (1.30–1.88), respectively. After adjusting for age, sex, DBP, WBC, CRP, and HOMA-IR, similar trends were observed. The fully adjusted ORs for low NKA of C3 and C4 were significantly higher compared with C1 (OR: 1.37, 95% CI: 1.08–1.75 for C3, and OR: 1.55, 95% CI: 1.20–2.00 for C4, respectively). In path analysis, RHR was shown to exert indirect effects on NKA upon consideration of the mediation effect of serum cortisol (**Supplementary Figure S1**).

**Table 3:** Odds ratios and 95% confidence intervals of low NKA according to resting heart rate categories.

	Category 1	Category 2	*p*	Category 3	*p*	Category 4	*p*	Overall *p*
		OR (95% CI)		OR (95% CI)		OR (95% CI)		
Model 1	1 (Ref.)	1.25 (1.05–1.50)	0.014	1.34 (1.12–1.60)	0.001	1.56 (1.30–1.88)	< 0.001	< 0.001
Model 2	1 (Ref.)	1.27 (1.06–1.52)	0.009	1.38 (1.16–1.66)	< 0.001	1.64 (1.36–1.98)	< 0.001	< 0.001
Model 3	1 (Ref.)	1.27 (1.00–1.61)	0.053	1.37 (1.08–1.75)	0.011	1.55 (1.20–2.00)	0.001	0.006

Model 1: Unadjusted.

Model 2: Adjusted for age and sex.

Model 3: Adjusted for age, sex, DBP, WBC, CRP, and HOMA-IR.

## 4 Discussion

This study demonstrated that a higher RHR is linked to low NKA. Individuals with elevated RHR exhibited poorer metabolic and inflammatory profiles, with the prevalence of low NKA being highest in those with higher RHR. Even after adjusting for various confounding factors, higher RHR remained significantly associated with greater odds of low NKA.

The analysis revealed significant differences in clinical characteristics among different RHR categories. Notably, individuals in the highest RHR category (≥80) had the poorest metabolic and inflammatory profiles, including higher DBP, WBC counts, CRP, insulin, and HOMA-IR, and lower IFN-γ levels suggesting that elevated RHR is a marker of both metabolic and cardiovascular risk. Previous studies have established a robust link between elevated RHR and metabolic syndrome (33, 34). Higher RHR was associated with increased metabolic syndrome prevalence and could predict its onset over time (33). Similarly, individuals with higher RHR were more likely to exhibit components of the metabolic syndrome, such as elevated blood pressure, dyslipidemia, and insulin resistance (34). The results of this study are consistent with these previous findings, as subjects with lower NKA showed significantly higher RHR, along with elevated WBC counts and CRP levels, indicating a state of chronic inflammation. This pro-inflammatory environment is known to contribute to insulin resistance and metabolic dysregulation, which are central to the development of metabolic syndrome. The association between RHR and inflammatory biomarkers is also well studied. For instance, Whelton et al. reported that higher RHR was linked to elevated levels of CRP, interleukin-6 (IL-6), and fibrinogen, markers that signify systemic inflammation (35). This inflammation can impair immune function, including NKA, potentially creating a feedback loop that exacerbates metabolic and cardiovascular risks.

Elevated RHR has been independently associated with increased cardiovascular and all-cause mortality, even after adjusting for inflammatory markers (36). The association of higher RHR with both low NKA and increased inflammatory markers in our study suggests that individuals with elevated RHR may be at a heightened risk for cardiovascular disease (CVD). Nanchen et al. found that higher RHR predicted incident heart failure and cardiovascular mortality in older adults, with inflammation and endothelial dysfunction playing significant roles (12). The link between NKA and CVD risk could be further explained by the role of chronic inflammation and immune dysfunction in atherosclerosis (37). Low NKA may reflect impaired immune surveillance, allowing for the progression of atherosclerotic plaques and increasing the likelihood of cardiovascular events.

The progressive increase in the prevalence of low NKA with higher RHR indicates a dose-response relationship, suggesting that elevated RHR correlates with a greater likelihood of impaired immune function. This relationship was further corroborated through multivariable logistic regression analysis, which confirmed that higher RHR categories are independently associated with increased odds of low NKA, even after adjusting for confounding variables. This robust association underscores the significance of elevated RHR as an independent predictor of reduced immune function. Path analysis identified a potential mechanism explaining the association between RHR and low NKA. Specifically, RHR was indirectly affecting NKA through elevated serum cortisol levels. This finding suggests that increased RHR may reflect heightened sympathetic nervous system activity, which is associated with stress and subsequently increased cortisol levels. While cortisol is essential for managing acute stress, chronically elevated levels due to prolonged stress can suppress immune function by activating the hypothalamic-pituitary-adrenal (HPA) axis (38).

Autonomic nervous system (ANS), one of the main homeostatic regulatory systems in the body, plays a crucial role in RHR. The two interacting branches of ANS, the sympathetic nervous system (SNS) and parasympathetic nervous system (PNS), must be in balance to maintain the physiological homeostasis (39). ANS balance is maintained through complex mechanisms involving central and peripheral regulation, reciprocal inhibition, and baroreceptor reflex (40). However, aging, acute and chronic stress, organic and idiopathic causes lead to ANS imbalance, characterized by increased SNS activity and reduced PNS activity at rest (41). Increased SNS activity releases norepinephrine and epinephrine, which bind to β1-adrenergic receptors on the pacemaker cells of SA node, activates intracellular signaling pathways that increase the production of cyclic adenosine monophosphate (cAMP), accelerates the depolarization of the pacemaker cells, and consequently increases RHR (42, 43). Stress can lead to ANS imbalance and increased heart rate, both of which are associated with suppressed NK cell activity and elevated pro-inflammatory cytokines (44). This supports the idea that stress-induced increases in RHR may compromise immune function. Suh et al. reported that elevated serum cortisol levels are associated with lower NKA, reinforcing the notion that chronic stress negatively impacts immune function through hormonal pathways (45). Also, Seiler et al. highlighted that everyday stressors can lead to chronic activation of the HPA axis, resulting in sustained high cortisol levels, which in turn can suppress immune function and reduce NKA (46). Moreover, Li et al. found that NK cell apoptosis is increased in conditions of oxidative stress, which is prevalent in chronic stress situations (47). This oxidative stress further contributes to the reduction in NKA observed in individuals with higher RHR. The balance of apoptosis and cell proliferation during heart development is crucial for normal cardiac function, and disruptions in these processes, potentially influenced by NKA, may impact RHR by altering the structural and functional integrity of the heart (48).

Our study has several limitations. First, this study is a cross-sectional design, which cannot reveal causality. Further longitudinal prospective studies are needed to confirm the predictive value of RHR for low NKA. Second, despite a relatively large sample size, our results have limited generalizability as our data were collected from a single hospital in South Korea. Third, lifestyle factors such as physical activity, medical conditions, and medications that could affect RHR and NKA were not fully evaluated. Fourth, RHR was measured by physician, not by echocardiogram. Lastly, the evaluation of NKA was done only using an IFN-γ assay. Our study did not incorporate techniques for directly quantifying NK cells or cytokines other than IFN-γ. Nevertheless, this new assay we employed has the advantage of simplicity, which can be more appropriate for large-scale studies. Despite these weaknesses, our study has the strength of being the first, to our knowledge, to demonstrate a significant association between high RHR and low NKA in a relatively large sample size. We also demonstrated a possible explanation for our results by showing the indirect effect of RHR on NKA upon consideration of the median effect of serum cortisol by path analysis.

In conclusion, this study found a significant link between higher RHR and reduced NKA, even after accounting for various confounding factors. Individuals with higher RHR had poorer metabolic and inflammatory profiles, with the lowest NKA levels seen in those with the highest RHR. Elevated RHR, which reflects increased sympathetic nervous system activity and stress, results in higher cortisol levels that suppress immune function. This finding underscores the importance of RHR as an independent predictor of reduced immune function and highlights its association with metabolic and cardiovascular risks. Thus, interventions aimed at lowering stress, RHR, and cortisol levels may help improve immune function and reduce these risks.

## Data Availability

The raw data supporting the conclusions of this article will be made available by the authors, without undue reservation.

## Supplementary material

Supplementary Figure 1 Path analysis to identify the direct and indirect effects of RHR on NKA upon consideration of the mediation effect of cortisol.
