# Supplementary figures and images for: Association between resting heart rate and low natural killer cell activity: a cross-sectional study
Source: Front Immunol. 2024 Sep 27;15:1465953. doi: 10.3389/fimmu.2024.1465953 (PMC11466811; doi:10.3389/fimmu.2024.1465953)

(A) Unadjusted

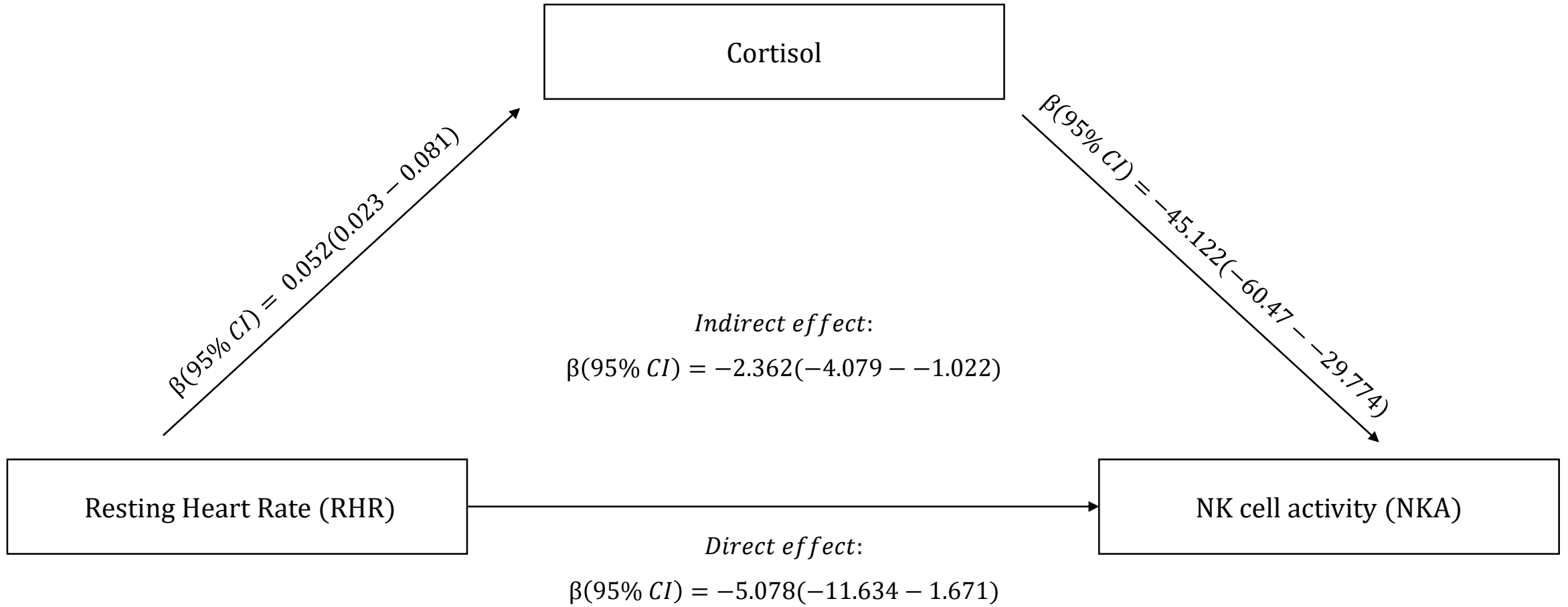

(B) Adjusted

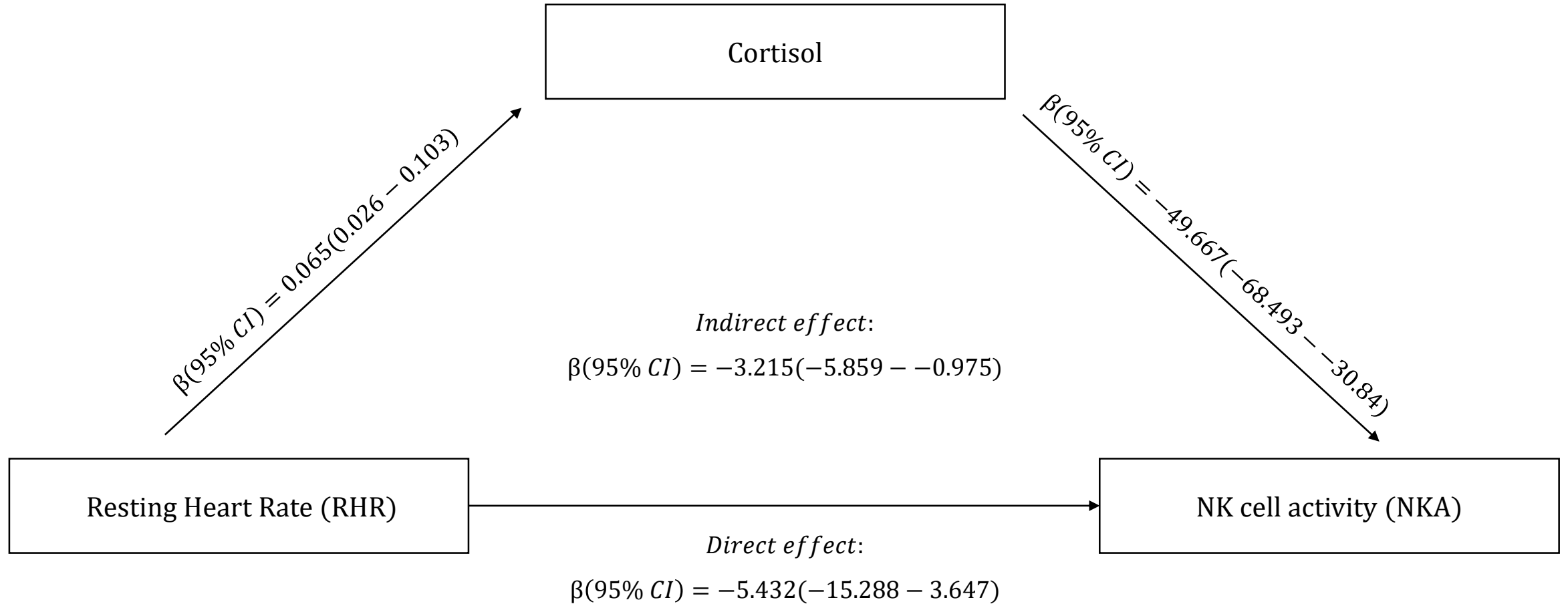

Supplement: Supplementary Figure 1 — Path analysis to identify the direct and indirect effects of RHR on NKA upon consideration of the mediation effect of cortisol. [file DataSheet1.pdf]
